# Fragility Score: a REMS-based indicator for the prediction of incident fragility fractures at 5 years

**DOI:** 10.1007/s40520-023-02358-2

**Published:** 2023-02-08

**Authors:** Paola Pisani, Francesco Conversano, Maurizio Muratore, Giovanni Adami, Maria Luisa Brandi, Carla Caffarelli, Ernesto Casciaro, Marco Di Paola, Roberto Franchini, Davide Gatti, Stefano Gonnelli, Giuseppe Guglielmi, Fiorella Anna Lombardi, Alessandra Natale, Valentina Testini, Sergio Casciaro

**Affiliations:** 1 Institute of Clinical Physiology, National Research Council, Lecce, Italy; 2 ASL-LE, Ospedale Vito Fazzi, Lecce, Italy; 3 Rheumatology Unit, University of Verona, Policlinico GB Rossi, Verona, Italy; 4 Italian Bone Disease Research Foundation (FIRMO), Florence, Italy; 5 Department of Medicine, Surgery and Neuroscience, University of Siena, Policlinico Le Scotte, Siena, Italy; 6 Department of Clinical and Experimental Medicine, Foggia University School of Medicine, Foggia, Italy; 7 R&D Department, Echolight Spa, Lecce, Italy

**Keywords:** Fragility Score, Radiofrequency Echographic Multi Spectrometry, REMS, Osteoporosis, Incident fragility fracture

## Abstract

**Background:**

Accurate estimation of the imminent fragility fracture risk currently represents a challenging task. The novel Fragility Score (FS) parameter, obtained during a Radiofrequency Echographic Multi Spectrometry (REMS) scan of lumbar or femoral regions, has been developed for the non-ionizing estimation of skeletal fragility.

**Aims:**

The aim of this study was to assess the performance of FS in the early identification of patients at risk for incident fragility fractures with respect to bone mineral density (BMD) measurements.

**Methods:**

Data from 1989 Caucasians of both genders were analysed and the incidence of fractures was assessed during a follow-up period up to 5 years. The diagnostic performance of FS to discriminate between patients with and without incident fragility fracture in comparison to that of the BMD T-scores measured by both Dual X-ray Absorptiometry (DXA) and REMS was assessed through ROC analysis.

**Results:**

Concerning the prediction of generic osteoporotic fractures, FS provided AUC = 0.811 for women and AUC = 0.780 for men, which resulted in AUC = 0.715 and AUC = 0.758, respectively, when adjusted for age and body mass index (BMI). For the prediction of hip fractures, the corresponding values were AUC = 0.780 for women and AUC = 0.809 for men, which became AUC = 0.735 and AUC = 0.758, respectively, after age- and BMI-adjustment. Overall, FS showed the highest prediction ability for any considered fracture type in both genders, resulting always being significantly higher than either T-scores, whose AUC values were in the range 0.472–0.709.

**Conclusion:**

FS displayed a superior performance in fracture prediction, representing a valuable diagnostic tool to accurately detect a short-term fracture risk.

## Introduction

Osteoporosis is a common skeletal disorder that causes reduced bone strength and increased risk of fragility fractures, occurring as a consequence of usually irrelevant low-energy traumas [1]. Osteoporosis-related fractures represent a significant burden for healthcare systems. As reported by several epidemiologic studies, fractures caused by osteoporosis occur to millions of people worldwide, with approximately 50% of women and 25% of men aged over 50 who are expected to suffer an osteoporosis-related fracture in their lifetime [2–4]. In 2013, it was estimated that almost 9 million fragility fractures (i.e., osteoporotic fractures) occur every year across the globe, more than a third of which in Europe, annually accounting for 2 million of disability-adjusted life years (DALYs) [5]. Two main factors contribute to the increasing burden of fragility fractures in the population: (1) growing diffusion of osteoporosis, which is a silent disease without any symptoms until a fracture occurs and whose incidence increases with population aging; (2) underdiagnosis and under-treatment of osteoporosis itself, which are in turn due to both a scarce awareness of the problem among the population and a lack of adequate techniques for accurate assessment of fracture risk [6–8].

Several risk assessment tools combining different risk factors have been developed, with various levels of complexity, but only a few were validated in population-based settings according to a proper methodological approach [9]. Among these, QFracture®, FRAX® and Garvan are mostly recommended for fracture prediction, with the last two methods taking into account also the bone mineral density (BMD) measurement, making the evaluation more accurate but also more complex [10]. However, none of the tools performed consistently better than the others [10] and no universal tool or guideline approach is currently able to meet the needs of every country worldwide [11].

Radiofrequency Echographic Multi-Spectrometry (REMS) is an innovative ultrasound technology that is applicable to the axial reference sites (i.e., lumbar vertebrae and femoral neck) for BMD assessment and fracture prediction [12–14]. It was found that the BMD T-score value measured by REMS was able to effectively estimate the fracture risk in the female population [14]. In this context, an additional REMS-based parameter, the Fragility Score (FS), has been implemented as a further indicator of skeletal fragility independent of BMD [15, 16].

According to the working principle of the REMS approach [17, 18], during an echographic scan of the femoral neck or lumbar vertebrae, the native raw unfiltered ultrasound signals backscattered by the target bone structures are automatically analysed. Following a comparison between the patient-specific spectral profiles with population-based anthropometrically-matched models of “fractured” and “non-fractured” subjects, FS value is obtained. This value corresponds to the percentage of analysed bone segments whose spectral features are more similar to those of a ‘‘fractured” bone model rather than to those of a ‘‘non-fractured” one [15, 16]. This parameter is independent of BMD and intrinsically related to the quality of bone microarchitecture, in agreement with the most recent research trends in this field, that are evolving towards the assessment of the actual bone strength independently of BMD [19, 20]. The first validation studies demonstrated a good correlation between FS and FRAX® results computed by including the BMD information: for the prediction of a major osteoporotic fracture, FS was found to closely correlate with the 10-year FRAX® fracture risk computed including the femoral neck BMD [15, 16].

This prospective study aims to assess the ability of FS to discriminate patients at risk of incident fragility fractures, including hip fracture or any other fracture, in a representative population and to evaluate its performance in comparison to REMS BMD T-score and DXA BMD T-score.

## Materials and methods

### Study design and enrollment

The clinical data were collected during a prospective observational study on female and male patients fulfilling the following eligibility criteria: Caucasian ethnicity, age in the range of 30–90 years, no significant walking impairments. The patients were recruited at the “Galateo” Hospital in San Cesario di Lecce (Lecce, Italy) from January 2016 to February 2019 and the follow-up ended in July 2021.

The enrolled patients were scanned at the lumbar spine and femoral neck both with DXA (according to their medical prescription) and REMS, resulting in the overall execution of 1989 lumbar scans, and 1812 femoral scans. The analysis of all DXA and REMS medical reports was performed separately for lumbar spine and femoral neck sites as well as for both genders.

The subsequent assessment of the possible incident fragility fractures relied on medical reports based on imaging investigations, such as radiographs, vertebral morphometry, etc., acquired during a follow-up period lasting up to 5 years. The enrolled patients were contacted every 6 months to assess their health status by telephonic interview and the actual nature of the possibly declared fractures was then verified as described. Traumatic fractures were excluded, whereas for patients suffering multiple fragility fractures, only the first occurred fracture was considered.

The study protocol was approved by the Ethics Review Board (ID: 2258/2011). All the enrolled patients voluntarily entered the study after signing the written informed consent.

### DXA and REMS acquisitions

DXA scans were performed according to the routine clinical procedures: spinal investigations were carried out with hip and knee flexed at 90°; for femoral scans, the patient’s femur was straight on the table, in a position such that the femoral shaft was parallel to the vertical edge of the obtained image, and with 15°–25° of internal rotation, which was achieved by using a dedicated positioning device. All the DXA medical reports were anonymized before the subsequent analyses.

REMS scans of lumbar vertebrae and proximal femur were performed employing the EchoStation device (Echolight S.p.a., Lecce, Italy), equipped with a convex transducer operating at the nominal frequency of 3.5 MHz and used as recommended by the manufacturer. For each completed acquisition, the final medical report, the corresponding sequence of B-mode images and the related unprocessed raw ultrasound signals were automatically stored. Lumbar spine scans were performed by placing the echographic transducer in a trans-abdominal position under the sternum, to initially visualize the L1 lumbar vertebra, and then moving it up to L4 according to the on-screen and audio indications provided by the device. Overall, a lumbar spine image acquisition lasted 80 s (20 s per vertebra). Proximal femur scans were performed by placing the echographic transducer parallel to the head-neck axis of the femur, to visualize the typical proximal femur profile, including the interfaces of the femoral head, neck and trochanter. Once the acquisition started, the operator simply held the image for 40 s, according to the indications provided by the device. For all the acquisitions (vertebral and femoral), transducer focus and scan depth were adjusted to have the target bone interface (i.e., vertebral surface or femoral neck) in the focal zone of the ultrasound beam and at a distance of at least 3 cm from the B-mode image bottom.

All the REMS medical reports, together with the corresponding echographic images and raw signals, were anonymized before starting the subsequent analyses.

### Data analysis

For the calculation of the FS, the ultrasound data acquired during an echographic scan was processed by an innovative algorithm that executed a series of spectral and statistical analysis on radiofrequency (RF) signals backscattered by the bone target, as previously described [15, 16]. Briefly, after automatic identification of the bone interface and the related region of interest (ROI), the spectral profiles of each patient were classified as ‘frail’ or ‘not-frail’ on the basis of a comparison with reference spectral models of fractured and not-fractured patients. In particular, the algorithm aimed at measuring the percentage of RF signal portions for which the spectral characteristics of the patients correlated with a frail bone whose data, collected in a proprietary database, derive from previous acquisitions on subjects who reported an osteoporotic fragility fracture. The database grouped all subjects from 30 to 90 years who were stratified into patient categories of 100 subjects each, where each category was based on distinct 5-year age intervals, BMI classes, and sex.

In more detail, the data analysis performed on individual patient datasets, involved the following steps:

1. Automatic segmentation of vertebral or femoral interfaces within the echographic images.
2. Automatic selection of an ROI for each segmented interface, including a specific RF signal segment for each scan line crossing the vertebral or femoral surface.
3. Classification of each RF segment as ‘‘frail” or ‘‘non-frail” on the basis of the correlation between its frequency spectrum and each of the two age-matched models stored in a previously built reference database.
4. For each considered ROI, calculation of the FS value, defined as the percentage of the analyzed RF portions that were classified as ‘‘frail” in the previous step.
5. Calculation of the FS value for the investigated patient as the average of all the ROI values.

The analysis was performed separately for patient gender and anatomical site: this means that female/male patients and the lumbar spine/femoral neck datasets were separately considered for the analysis. Lumbar spine datasets were integrated with the data of a generic major osteoporotic fracture (occurring at spine, hip, radius, humerus or forearm), while femoral datasets were integrated with the corresponding data of possible hip fractures only. The differences between the patients who suffered a fragility fracture during the follow-up period and those who did not were investigated in terms of the median and interquartile range (IQR), and the statistical differences were assessed by non-parametric Mann–Whitney test. The discriminative ability of FS in the identification of the patients prone to incident fragility fractures was investigated through Receiver Operating Characteristic (ROC) curves, assessing also the optimal cut-off value, which represents the best tradeoff between sensitivity and specificity of any diagnostic tool. Sensitivity, specificity, positive predictive value (PPV), negative predictive value (NPV) and odds ratio (OR) were calculated in correspondence with the identified cut-off value (MedCalc® Software, version 20.104).

The discriminative ability of FS was compared with the ones of BMD T-score values obtained through either DXA or REMS scans. Area under the curve and OR for BMD T-score values were calculated in correspondence with the conventional cut-off value used for the diagnosis of osteoporosis (i.e. T-score ≤ −2.5). ROC curve adjustment for the main potentially influencing covariates (age and body mass index (BMI) between patients with and without incident fragility fractures) were performed through linear fitting using RStudio (version 4.1.2) [21]. The differences between ROC curves evaluating the discriminative ability of the considered parameters were compared to each other using De Long test [22], with p-values considered significant if below the threshold of 0.05.

### Intra- and inter-operator repeatability assessment

Intra- and inter-operator repeatability of FS measurements were assessed using data of repeated REMS scans on each considered anatomical site from the first enrolled patients, following the method described by Di Paola et al. [12].

According to ISCD, intra-operator repeatability (defined as “short-term precision” [23]), was assessed by performing three repeated scans of the spine and femur on each patient by an experienced operator, to determine the best achievable precision. For each considered anatomical site, precision was assessed on the measurements acquired on a set of 15 patients (6 males; 9 females) and a total of 45 cases was included in the analysis.

Analogously, inter-operator repeatability was assessed by performing three repeated scans at the spine and femur on each patient by three different operators, among whom one experienced and two newly trained, with the aim to evaluate the degree of variability of the obtained results. For each considered anatomical site, inter-operator repeatability was determined on the measurements acquired on a set of 15 patients (6 males; 9 females) and a total of 45 cases was included in the analysis.

As recommended by the International Society of Clinical Densitometry (ISCD) guidelines and following previous approaches for BMD precision [24], both intra- and inter-operator repeatability were calculated as the root-mean-square coefficient of variation (RMS-CV) at 95% confidence level (available at http://www.iscd.org/resources/ calculators/).

## Results

### Patients’ characteristics

Concerning lumbar spine acquisitions, of the 1989 patients who were enrolled, during the follow-up period, 185 patients (9.3%) voluntarily dropped out from the study or died, therefore 1804 patients completed the study. Of these patients, 1289 were women and 515 were men. Correspondingly, of the 1812 patients who were considered for femoral neck analysis, 133 patients (7.3%) voluntarily dropped out from the study or died. Of the 1679 patients who completed the follow-up, 1205 were women and 474 were men. Considering the overall lumbar spine dataset, after a mean follow-up of 3.5 years (range 2–5), 248 patients (13.7%) suffered from an incident fracture, whereas, for the femoral neck dataset, after a mean follow-up of 3.3 years (range 2–5), 48 patients (2.9%) suffered from an incident hip fracture. Table 1 summarizes the main patients’ characteristics, stratified by gender for each considered anatomical site. Similarly to DXA and REMS-measured mean BMD T-scores, FS values resulted to be significantly different in the fractured group compared to the non-fractured counterpart in both genders and anatomical regions.

**Table 1:** The characteristics of the enrolled patients are presented for both women and men and for each considered anatomical site. The subgroups of patients with and without incident fragility fracture are referred to as “fractured patients” and “non-fractured patients”, respectively. Results are reported as median value and interquartile range in brackets. *P*-values refer to the comparison between patients with and without incident fragility fracture

Female population	Overall dataset	Fractured patients	Non-fractured patients	*p*-value
Lumbar spine				
Number of patients	1289	181	1108	N.A
Age (years)	60.0 (54.0–66.0)	71.0 (63.0–74.0)	59.0 (54.0–64.0)	< 0.0001
Height (cm)	160.0 (155.0–165.0)	158.0 (155.0–162.3)	160.0 (156.0–165.0)	< 0.0001
Weight (kg)	62.0 (57.0–70.0)	61.0 (57.0–70.0)	62.0 (57.0–70.0)	0.732
BMI (kg/m^2^)	24.4 (22.3–26.6)	25.0 (22.9–27.0)	24.2 (22.2–26.6)	0.039
DXA T-score	−2.1 (−2.9 to −1.1)	−2.8 (−3.4 to −2.0)	−1.9 (−2.7 to −1.0)	< 0.0001
REMS T-score	−2.1 (−2.9 to −1.1)	−2.9 (−3.6 to −2.0)	−2.0 (−2.8 to −1.0)	< 0.0001
Fragility Score	31.2 (25.9–39.2)	53.0 (34.8–61.8)	29.9 (25.6–36.2)	< 0.0001
Femoral neck				
Number of patients	1205	30	1175	N.A
Age (years)	62.0 (55.0–68.0)	72.0 (67.0–80.0)	62.0 (55.0–68.0)	< 0.0001
Height (cm)	160.0 (155.0–165.0)	158.0 (153.0–165.0)	160.0 (155.0–165.0)	0.35
Weight (kg)	63.0 (58.0–70.0)	64.5 (60.0–69.0)	63.0 (58.0–70.0)	0.68
BMI (kg/m^2^)	24.7 (22.5–27.1)	24.5 (23.4–27.9)	24.7 (22.5–27.1)	0.21
DXA T-score	−1.8 (−2.4 to −1.2)	−2.0 (−2.8 to −1.5)	−1.8 (−2.4 to −1.2)	0.028
REMS T-score	−1.9 (−2.4 to −1.1)	−2.4 (−2.9 to −1.6)	−1.9 (−2.4 to −1.1)	0.0102
Fragility Score	25.3 (21.2–33.1)	41.0 (29.0–54.8)	25.2 (21.1–32.6)	< 0.0001

Male population	Overall dataset	Fractured patients	Non-fractured patients	*p*-value
Lumbar spine				
Number of patients	515	67	448	N.A
Age (years)	62.0 (48.3–73.0)	72.0 (57.3–78)	60.5 (47.0–71.0)	< 0.0001
Height (cm)	173.0 (167.3–178.0)	168.0 (162.0–173.8)	174.0 (169.0–179.0)	< 0.0001
Weight (kg)	77.0 (70.0–85.0)	74.0 (62.0–78.8)	77.5 (70.0–85.5)	< 0.0001
BMI (kg/m^2^)	25.7 (23.4–28.1)	25.1 (22.8–28.1)	25.7 (23.5–28.1)	0.31
DXA T-score	−0.8 (−1.8 to 0.1)	−1.8 (−2.5 to 0.0)	−0.8 (−1.6 to 0.1)	0.0004
REMS T-score	−0.9 (−1.9 to 0.1)	−1.7 (−2.7 to −0.2)	−0.8 (−1.7 to 0.1)	0.0001
Fragility Score	24.1 (20.9–31.7)	43.6 (26.8–58.3)	23.6 (20.8–28.8)	< 0.0001
Femoral neck				
Number of patients	474	18	456	N.A
Age (years)	60.0 (48.0–69.0)	75.0 (61.0–81.0)	60.0 (48.0–69.0)	0.0038
Height (cm)	172.0 (167.0–178.0)	169.5 (162.0–174.0)	173.0 (167.0–178.0)	0.017
Weight (kg)	77.0 (69.0–85.0)	68.0 (58.0–79.0)	77.0 (70.0–85.0)	0.014
BMI (kg/m^2^)	26.0 (23.5–28.4)	24.5 (22.0–27.3)	26.0 (23.5–28.4)	0.13
DXA T-score	−1.1 (−1.8 to −0.3)	−1.9 (−2.6 to −0.6)	−1.0 (−1.7 to −0.3)	0.011
REMS T-score	−1.2 (−1.8 to −0.3)	−2.2 (−2.7 to −0.9)	−1.1 (−1.8 to −0.3)	0.0009
Fragility Score	19.5 (13.0–33.4)	47.9 (25.0–53.8)	19.0 (12.9–32.2)	< 0.0001

*BMI* Body mass index, *DXA* dual-energy X-ray absorptiometry, *REMS* Radiofrequency Echographic Multi Spectrometry, *N.A.* not available

### Performance of Fragility Score in the prediction of fragility fractures

The area under the curve (AUC) obtained by the lumbar spine FS for the discrimination between female patients with and without an incident fragility fracture was 0.811 (*p* < 0.0001) (Table 2). When the best cut-off value of FS = 37.2 was considered, the fracture risk estimation was associated with an OR = 9.23 (95%CI 6.47–13.17, *p* < 0.0001), in correspondence of which sensitivity and specificity were 72.4% and 77.9% (PPV = 34.8%, NPV = 94.5%), respectively. The AUC obtained considering the FS derived from femoral neck analysis for the discrimination between women with and without incident hip fractures was 0.780 (*p* < 0.001) (Table 2). Sensitivity and specificity values of 70% and 73.2% (PPV = 6.3%, NPV = 99.0%), respectively, corresponded to the best cut-off value (FS = 31.9), which was associated with an OR = 6.37 (95%CI 2.89–14.06, *p* < 0.0001).

**Table 2:** Area under the curve of Fragility Score (FS) at the lumbar spine (LS) and femoral neck (FEM) for the prediction of fragility fractures in the female and male populations

	FS AUC	*P*-value	Cut-off	Sensitivity	Specificity
Women (LS)	0.811	< 0.0001	37.2	72.4%	77.9%
Women (FEM)	0.780	< 0.0001	31.9	70.0%	73.2%
Men (LS)	0.780	< 0.0001	30.2	71.6%	79.0%
Men (FEM)	0.809	< 0.0001	32.6	72.2%	76.1%

Concerning the male dataset, the AUC obtained by the lumbar spine FS was 0.780 (*p* < 0.0001) (Table 2). In correspondence of lumbar spine FS = 30.2 (best cut-off), a sensitivity of 71.6% and a specificity of 79.0% (PPV = 33.8%, NPV = 94.9%) were obtained, with a corresponding OR = 9.51 (95%CI 5.34–16.96, *p* < 0.0001). The AUC obtained by the femoral neck FS for the discrimination between male patients with and without incident fracture at the hip was 0.809 (*p* < 0.0001), with an optimal cut-off of 32.6, corresponding to a sensitivity of 72.2%, a specificity of 76.1% (PPV = 10.7% and NPV = 98.6%) and an OR = 8.28 (95%CI 2.89–23.73, *p* = 0.0001) (Table 2).

### Comparison between Fragility Score and BMD T-scores for the prediction of fragility fractures

The performance of FS in fracture prediction was compared to that of the DXA and REMS BMD T-scores for both women and men at the two anatomical sites.

Considering the lumbar spine investigations in the female group, in correspondence of T-score ≤ −2.5 (which is the conventional cut-off value used for osteoporosis diagnosis), the OR derived from REMS BMD T-score was 3.58 (95%CI 2.57–4.99, *p* < 0.0001), whereas the OR for DXA BMD T-score was 2.50 (95%CI 1.81–3.44, *p* < 0.0001). The AUCs obtained by the lumbar spine REMS BMD T-score and DXA BMD T-score for the discrimination between women with and without incident fragility fractures were 0.709 and 0.678, respectively, and these values were both significantly lower than the AUC obtained by the lumbar spine FS (AUC = 0.811) (*p* = 0.003 and *p* < 0.0001, respectively).

**Fig. 1:**
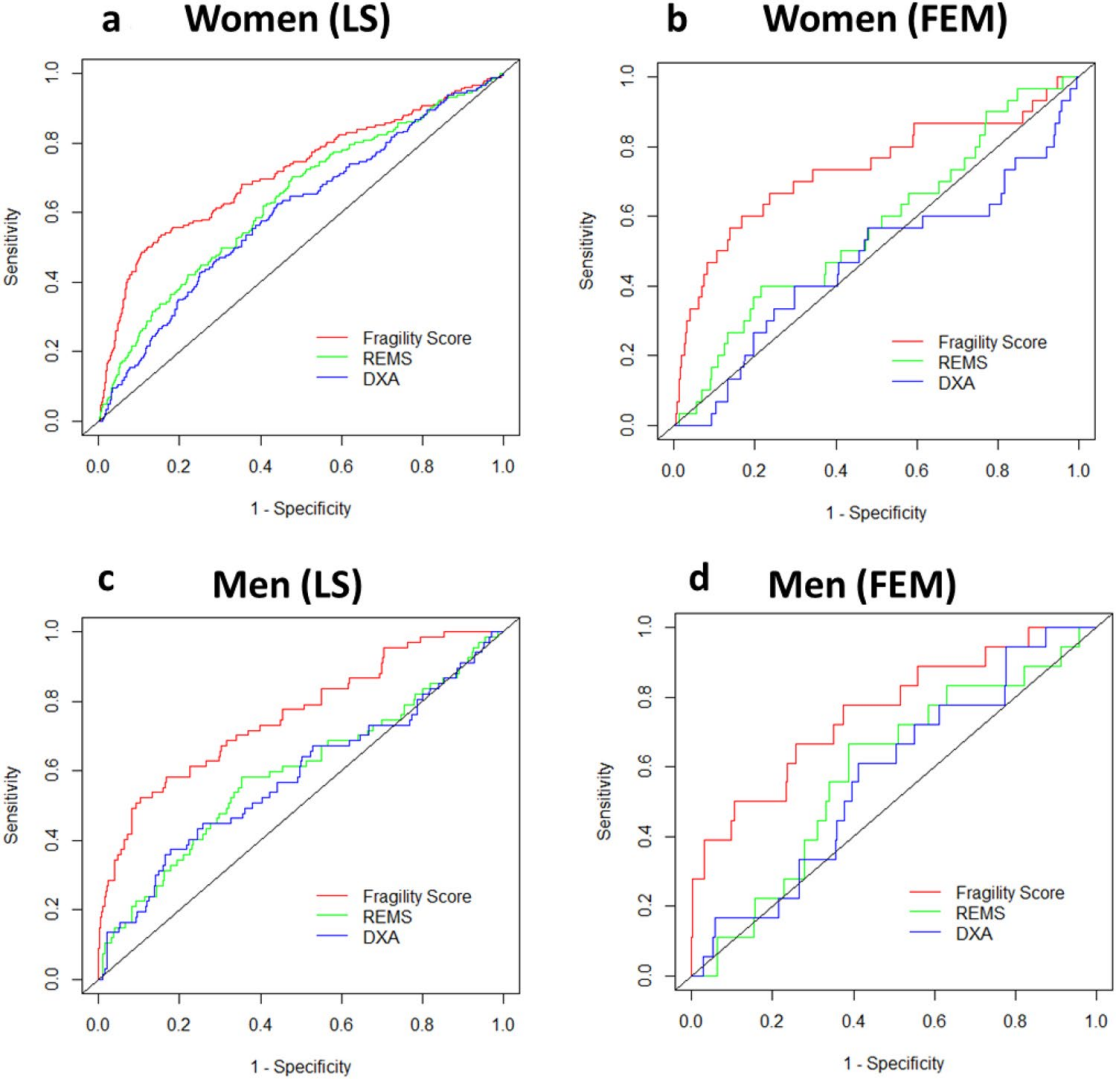
Comparison of age- and BMI-adjusted ROC curves for men and women at the lumbar spine (LS) and femoral neck (FEM). ROC curves showing sensitivity and specificity of REMS BMD T-score (green), DXA BMD T-score (blue) and Fragility Score (red) adjusted for age and body mass index (BMI), with a grey diagonal line representing the null hypothesis (area under the curve, AUC = 0.5)

For the same group, the ROC analysis adjusted for age found that the AUC for lumbar FS, REMS BMD T-score and DXA BMD T-score were 0.713, 0.622 and 0.590, respectively (Table 3), and the differences among all the AUCs were statistically significant (*p* = 0.01 and *p* = 0.0004, respectively versus FS). When adjusting for both age and BMI, FS performed the best with an AUC of 0.715, which was higher than both REMS- and DXA-measured T-score values, whose AUCs were 0.636 (*p* = 0.02) and 0.603 (*p* = 0.001), respectively (Fig. 1a).

**Table 3:** Area Under the Curve (AUC) of Fragility Score (FS), REMS BMD T-score and DXA BMD T-score at the lumbar spine (LS) and femoral neck (FEM) for the prediction of fragility fractures in the female and male populations

	Age-adjusted AUC	*P*-value			Age- and BMI- adjusted AUC	*P*-value		
		REMS T-score vs FS	DXA T-score vs FS	DXA vs REMS T-score		REMS T-score vs FS	DXA T-score vs FS	DXA vs REMS T-score
Women (LS)								
FS	0.713	0.01	0.0004	0.001	0.715	0.02	0.001	0.001
REMS T-score	0.622				0.636			
DXA T-score	0.590				0.603			
Women (FEM)								
FS	0.739	0.03	0.02	0.04	0.735	0.05	0.0003	0.37
REMS T-score	0.557				0.568			
DXA T-score	0.524				0.472			
Men (LS)								
FS	0.726	0.02	0.008	0.23	0.758	0.0007	0.0002	0.28
REMS T-score	0.594				0.592			
DXA T-score	0.560				0.579			
Men (FEM)								
FS	0.764	0.02	0.007	0.97	0.758	0.001	0.001	0.68
REMS T-score	0.617				0.589			
DXA T-score	0.616				0.575			

Dual-energy X-ray absorptiometry (DXA); Radiofrequency Echographic Multi Spectrometry (REMS)

Concerning the femoral investigations for the female population, using the conventional cut-off (T-score ≤ −2.5) to discriminate between patients with and without incident hip fractures, OR = 3.40 (95%CI 1.64–7.05, *p* = 0.001) was obtained for REMS BMD T-score, in contrast to OR = 2.79 (95%CI 1.34–5.81, *p* = 0.006) estimated by the DXA BMD T-score. Correspondingly, it was found that the AUC values for REMS BMD and DXA BMD T-score were 0.637 and 0.611, respectively, and these values were significantly lower than that obtained by the femoral neck FS (AUC = 0.780), (*p* = 0.03 and *p* = 0.02, respectively). Analogously, for the femoral investigation in the female population, the age-adjusted AUC of the FS was the highest (0.739), against the AUC found for the REMS BMD T-score and DXA BMD T-score, which were equal to 0.557 (*p* = 0.03) and 0.524 (*p* = 0.02) respectively, when discriminating between patients with and without incident hip fractures (Table 3). Age- and BMI-adjusted FS AUC yielded 0.735 in comparison to REMS BMD T-score and DXA BMD T-score of 0.568 (*p* = 0.05) and 0.472 (*p* = 0.0003), respectively (Fig. 1b).

With respect to the lumbar spine dataset for the male population, considering as usual the T-score threshold of −2.5, REMS BMD T-score yielded OR = 5.81 (95%CI 3.31–10.21, *p* < 0.0001), as opposed to OR = 3.65 (95%CI 1.95–6.84, *p* = 0.0001) obtained by DXA BMD T-score. The AUCs obtained by the lumbar spine REMS and DXA BMD T-scores for the discrimination between patients with and without incident fragility fractures were 0.652 and 0.635, respectively, whereas FS-derived AUC resulted to be significantly higher than those obtained by both T-scores (AUC = 0.780; *p* = 0.007 and *p* = 0.002, respectively). When correcting the ROC curves for age, the AUCs of lumbar FS, REMS BMD T-score and DXA BMD T-score were 0.726, 0.594, 0.560 respectively, and the superior discriminative ability of the FS with respect to both T-scores was statistically significant (*p* = 0.02 and *p* = 0.008, respectively) (Table 3). When adjusting for both age and BMI, FS yielded an AUC of 0.758, which was significantly higher than that of both REMS- and DXA-measured BMD T-scores, whose AUCs were 0.592 (*p* = 0.0007) and 0.579 (*p* = 0.0002), respectively (Fig. 1c).

Concerning the femoral neck assessment in the male population, when the conventional cut-off T-score value was considered, OR = 5.96 (95%CI 2.20–16.15, *p* = 0.0005) and OR = 4.21 (95%CI 1.30–13.60, *p* = 0.02) were obtained for REMS and DXA BMD T-score values, respectively. The AUCs of the femoral neck REMS and DXA BMD T-score to discriminate between male patients with and without fractures at the hip were 0.671 and 0.669, respectively, which were significantly lower compared to the AUC obtained by the femoral neck FS (0.809) (*p* = 0.03 and *p* = 0.008, respectively). ROC analysis adjusted for age at the femoral neck in males identified an FS-related AUC of 0.764, which was significantly higher than the AUCs obtained by both the T-score values (AUC = 0.617 for REMS BMD T-score with *p* = 0.02 and AUC = 0.616 for DXA BMD T-score with *p* = 0.007) (Table 3). Similarly, adjustment for both age and BMI produced an AUC of 0.758 for FS, which was significantly higher than that of both REMS- and DXA-measured BMD T-scores, whose AUCs were 0.589 (*p* = 0.001) and 0.575 (*p* = 0.001), respectively (Fig. 1d).

### Intra- and inter-operator repeatability assessment

Intra-operator repeatability (precision) of FS, expressed as RMS-CV, was 0.49% for the lumbar spine and 0.43% for a femoral neck at 95% confidence level. Analogously, inter-operator repeatability produced the following results: RMS-CV = 0.73% for the lumbar spine and RMS-CV = 0.64% for the femoral neck.

## Discussion

Currently, there is a large interest in the identification of parameters and technologies that might predict the occurrence of fragility fractures in a clinically sustainable framework [19, 27]. This need is mostly driven by the enormous economic and societal burden of fragility fractures, which are expected to rise because of the increasing life longevity of the population. On the other side, such burden impacts the autonomy of these individuals, being estimated that 50% of patients with hip fracture lose the ability to lead an independent life [28].

The present study assessed the effectiveness of the FS measured by REMS in the identification of patients prone to fragility fractures over a 5-year period. Measurements were performed both at the lumbar spine and femoral neck, which represent the reference anatomical sites typically considered for the diagnosis of osteoporosis. The performance obtained by FS was compared with that of the BMD T-score, measured with either REMS or DXA, with the latter being the clinically recognized parameter for the diagnosis of osteoporosis and the main clinically available indicator to predict the fracture risk [19, 28]. With the present study, the superior predictive capacity of the FS compared to the BMD T-score values for the identification of patients at risk for incident fragility fractures has been demonstrated. In particular, for women, based on the reported OR values, the lumbar spine FS above the 37.2 cut-off identified a ninefold higher risk for fragility fracture, considerably superior to the risk predicted using any BMD T-score (i.e. in this study, it was found a 2.5 times higher risk for osteoporotic patients diagnosed by DXA BMD T-score and 3.6 times higher risk for osteoporotic patients diagnosed by REMS BMD T-score). Consistent results were obtained for femoral neck FS, where women with values above 31.9 showed a 6 times higher risk of hip incident fracture, demonstrating greater effectiveness of FS in estimating hip fracture prediction compared to either T-scores. Concerning the male population, the FS cut-off values identified in this study ascertained that men had about 9.5-fold increased risk of fragility fracture using the lumbar FS and 8.3-fold increased risk of the hip fracture using the femoral FS. Both lumbar and femoral FS values predicted a higher risk than that identified with the conventional −2.5 BMD T-score threshold.

It is important to note that the predicted risk of fracture observed for DXA BMD T-score was in line with that observed in previous studies on large female populations [29–31], as well as for REMS BMD T-score [14]. Concerning the assessment of fracture risk in a male population, the literature-based evidence about the relationship between BMD and fracture risk is more limited than for the female population but, overall, the reported risk gradients are similar between both genders [30]–[32]. Interestingly, although fragility is naturally correlated with age, the reported age-adjusted results have shown that FS adds unique information peculiarly related to bone quality and only marginally associated with age or other anthropometric factors that might act as confounding variables.

The results obtained from the adjusted FS AUC are within a similar range as that estimated for the main clinically used tool for the 10-year prediction of fracture risk in clinical practice, FRAX®. Indeed, a meta-analysis by Marques and colleagues has shown that the AUCs obtained by FRAX® in the identification of women at risk of osteoporotic fractures and hip fractures were 0.65 and 0.74, respectively, whereas for men it reached 0.63 and 0.71, respectively [33]. When FRAX® was associated with the BMD information, the AUC marginally increased to 0.67 and 0.79 for the identification of hip and major osteoporotic fractures in women, respectively [33]. Clearly after integrating the BMD, FRAX®-based predictions of hip fractures appeared to be more accurate, with values comparable to those obtained by FS in the current study. In the same study, when predictions for major osteoporotic fractures were considered, the FS showed a much higher discriminative ability compared to FRAX® assessments either with and without BMD in both women and men. The latter results were consistent with another meta-analysis [10] that reported AUCs in the range 0.64–0.72 for Garvan and FRAX®, both integrating the DXA-measured BMD, therefore confirming a similar or even superior performance of FS in comparison to those alternative predictive tools.

It is noteworthy to mention that, compared to the abovementioned predictive tools that rely on a 10-year fracture risk estimation, the FS captures the imminent fracture risk in a shorter timeframe. Since it is reported that the risk of refracture following an initial fracture is particularly high within the following 2 years [34], FS can be useful to prevent repeated fractures and, on the other hand, to promote immediate interventions for those who, although never fractured, present an increased skeletal frailty that makes them at very high risk of fracture in the short-term. Furthermore, the adoption of FS in clinical practice might allow to overcome the principal shortcomings of conventional densitometry when used for fracture risk estimation, thanks to the absence of ionizing radiation and to the reduced costs that make its use suitable also for widespread population screening. Despite DXA makes use of the −2.5 BMD T-score threshold as an intervention criterion for fracture risk prevention, it is widely known that several fractures occur with less severe bone density losses [5, 11, 30, 35]. Clearly, the development of more effective risk assessment tools still represents an unmet clinical need [19].

Moreover, this study confirms the suitability of FS in a clinical setting for short-term monitoring of bone health. The low precision errors demonstrated here by the precision (RMS-CV = 0.49% for lumbar spine and RMS-CV = 0.43% for femoral neck) and repeatability study (RMS-CV = 0.73% for lumbar spine and RMS-CV = 0.64% for femoral neck), implies that FS is able to detect minimal skeletal changes, not attributable to instrument measurement errors nor operator experience.

However, this study is limited by data on bone fragility that does not consider other clinical factors that predict the risk of fractures, including the history of previous fractures, parental history, secondary osteoporosis, alcohol consumption and medication use. Future studies are warranted to combine the FS with all these BMD-independent predictors of osteoporosis-related fractures.

Despite these shortcomings, increasing scientific evidence has already demonstrated the clinical validity of the REMS technology in capturing skeletal fragility associated with bone alterations [36, 37]. In particular, the REMS-based T-score was able to measure a significantly lower BMD value in comparison to the DXA BMD T-score in fractured patients with secondary osteoporosis caused by diabetes and anorexia [38, 39]. In patients living with chronic kidney disease who are at increased risk of fragility fractures, REMS has recently been demonstrated not to be influenced by an artefactual increase of lumbar BMD due to the presence of aortic calcifications [40]. Additionally, REMS investigation in subjects affected by rheumatoid arthritis demonstrated a major risk of osteoporosis as well as a higher fracture risk compared to the control counterpart [41]. Recently, FS was found to effectively discriminate between non-fractured and fractured patients with primary and disuse-related osteoporosis, having the latter a more compromised skeletal fragility [42]. For this reason, this technique has been acknowledged in the Guidelines by the National Institute of Health as a valid diagnostic alternative for the management of osteoporosis diagnosis, risk stratification and continuity of care of fragility fractures [43].

## Conclusion

This prospective study demonstrated that FS is a reliable predictor of 5-year fracture probability in a representative population including both female and male subjects. The reported results showed that FS enables effective identification of patients at risk for a generic major osteoporotic fracture (occurring at the spine, hip, radius, humerus or forearm) upon lumbar investigations, as well as the discrimination of patients at specific risk for hip fractures following a femoral assessment. This newly developed diagnostic indicator could help predicting imminent fracture risk in the short term, exploiting specific FS-based intervention thresholds. This approach would benefit not only patients at risk of re-fracture to timely predict the occurrence of a second fracture, but it would also improve the early identification of high-risk categories to prevent the occurrence of the first fracture.

## Data Availability

All relevant data will be freely available to any researcher, upon a direct request and under a dedicated agreement to the corresponding author.
